# Clinical efficacy and safety of robotic retroperitoneal lymph node dissection for testicular cancer: a systematic review and meta-analysis

**DOI:** 10.3389/fonc.2023.1257528

**Published:** 2023-12-19

**Authors:** Yacheng Yuan, Dawei Zhang, Yiping Ning, Hengfeng Luo, Xiaolong Qiu, Yangyang Tan, Yuxiang Li, Xukai Yang

**Affiliations:** ^1^ Department of Urology, The 940 Hospital of Joint Logistics Support Force of Chinese PLA, Lanzhou, China; ^2^ The First Clinical Medical College of Gansu University of Chinese Medicine, Lanzhou, China; ^3^ Department of Urology, Central Hospital of Gansu Province, Lanzhou, China

**Keywords:** retroperitoneal lymph node dissection, robotics, R-RPLND, testicular cancer, meta-analysis

## Abstract

**Background:**

Retroperitoneal lymph node dissection (RPLND) is an effective treatment for testicular tumors. In recent years, with the development of robotics, many urological procedures performed via standard laparoscopy have been replaced by robots. Our objective was to compare the safety and efficacy of robotic retroperitoneal lymph node dissection (R-RPLND) versus Non-robotic retroperitoneal lymph node dissection (NR-RPLND) in testicular cancer.

**Methods:**

Pubmed, Embase, Scopus, Cochrane Library, and Web of Science databases were searched for literature on robotic surgery for testicular germ cell tumors up to April 2023. The statistical and sensitivity analyses were performed using Review Manager 5.3. Meta-analysis was performed to calculate mean difference (MD), odds ratio(OR), and 95% confidence interval (CI) effect indicators.

**Results:**

Eight studies with 3875 patients were finally included in this study, 453 with R-RPLND and 3422 with open retroperitoneal lymph node dissection (O-RPLND)/laparoscopic retroperitoneal lymph node dissection (L-RPLND). The results showed that R-RPLND had lower rates of intraoperative blood loss (MD = -436.39; 95% CI -707.60 to -165.19; P = 0.002), transfusion (OR = 0.06; 95% CI 0.01 to 0.26; P = 0.0001), total postoperative complication rates (OR = 0.39; 95% CI 0.21 to 0.70; P = 0.002), and length of stay (MD=-3.74; 95% CI -4.69 to -2.78; P<0.00001). In addition, there were no statistical differences between the two groups regarding perioperative and oncological outcomes regarding total operative time, the incidence of postoperative complications grade≥III, abnormal ejaculation rate, lymph node yield, and postoperative recurrence rate.

**Conclusions:**

The R-RPLND and O-RPLND/L-RPLND provide safe and effective retroperitoneal lymph node dissection for testicular cancer. Patients with R-RPLND have less intraoperative bleeding, shorter hospitalization period, fewer postoperative complications, and faster recovery. It should be considered a viable alternative to O-RPLND/L-RPLND.

**Systematic Review Registration:**

https://www.crd.york.ac.uk/PROSPERO, identifier CRD42023411696.

## Introduction

Testicular germ cell tumors (TGCT) represent the most prevalent solid neoplasms in males aged 20 to 44 years. There has been a persistent rise in the incidence of both seminomatous and non-seminomatous subtypes of TGCT during the past two decades (1). Retroperitoneal lymph node dissection (RPLND) is usually used as an effective treatment for testicular germ cell tumors, primarily in patients with clinical stage I and II non-seminomatous germ cell tumors (NSGCT) and those who present with residual masses after chemotherapy (2, 3). In addition, recent pilot studies have shown that RPLND may also be a therapeutic option for testicular seminomas with clinically low-volume retroperitoneal lymphadenopathy (4).

Open retroperitoneal lymph node dissection (O-RPLND) is the preferred method for retroperitoneal surgical management of NSGCT. Nevertheless, compared to other modalities, the heightened destructiveness of this procedure can contribute to notable postoperative complications and an extended hospital stay (5). In primary clinical stage I and II NSGCT, minimally invasive RPLND has emerged as an appealing alternative to O-RPLND, exhibiting promising initial oncological outcomes alongside a reduced complication rate (6, 7). The advent of laparoscopic retroperitoneal lymph node dissection (L-RPLND) was first reported in 1994, showcasing characteristics such as a quicker postoperative recovery period, diminished blood loss, and a lower frequency of complications compared to O-RPLND (8). However, the long-term oncological outcomes of L-RPLND have yet to be studied as rigorously as those of O-RPLND, and the learning curve for this procedure is very steep (9, 10). The relatively short duration of follow-up for L-RPLND, along with concerns surrounding the need for postoperative chemotherapy in cases of positive masses and the potential compromise of lymph node yield due to incomplete clearance of large vessels, has prompted researchers to express certain reservations (11). The natural progression within the minimally invasive urological oncology surgery field from laparoscopic to robotic techniques set the stage for the pioneering use of robotic retroperitoneal lymph node dissection (R-RPLND) by Davol et al. (12) in 2006. The advantages of R-RPLND, including a shorter learning curve, three-dimensional visualization, and enhanced maneuverability of instruments, have positioned it as a promising option for the management of clinical stage I and II NSGCT, as well as post-chemotherapy RPLND (13, 14). However, there remains ongoing debate regarding the safety and efficacy of R-RPLND in the context of testicular cancer (15). To gain further insights into the clinical value of R-RPLND for testicular germ cell tumors, especially in NSGCT, this study aims to conduct a systematic review and meta-analysis, providing a comprehensive analysis and evaluation of available literature on the subject.

## Materials and methods

The present investigation adheres to the guidelines set forth by the Preferred Reporting Items for Systematic Reviews and meta-Analyses (PRISMA) (16). We have duly registered this study with PROSPERO, and the registration number is (2023 CRD42023411696).

### Search strategy

A meticulous and comprehensive systematic search was carried out across multiple databases, including PubMed, Embase, Scopus, Cochrane Library, and Web of Science, encompassing publications until April 2023. The search strategy utilized the following search terms: ((((robotic) OR (robotics)) OR (robots)) AND ((retroperitoneal lymph node dissection) OR (RPLND))) AND (((testicular cancer) OR (testicular neoplasms)) OR (testicular tumor)). Additionally, a manual search of the references of identified articles was conducted to ensure a thorough scope, irrespective of language or publication year.

### Inclusion and exclusion criteria

Inclusion criteria: (i) inclusion of patients who underwent retroperitoneal lymph node dissection for the treatment of testicular cancer; (ii) consideration of randomized controlled trials that compared the efficacy and safety of robotic retroperitoneal lymph node dissection (R-RPLND) with non-robotic approaches (O-RPLND/L-RPLND), as well as prospective or retrospective cohort studies; (iii) evaluation of relevant outcomes such as operative time, estimated blood loss, transfusion rate, operative complications, total complications, abnormal ejaculation, lymph node yield, length of hospital stay, and postoperative recurrence; and at least one of these outcome indicators must have been observed for measurement within the study; (iv) inclusion of publications in the English language.

Exclusion criteria: (i) unavailability of accurate data; (ii) duplicate publications; (iii) comparison with other surgical techniques; (iv) reviews, comments, letters, conference abstracts, and case reports.

### Data extraction and quality assessment

Two independent researchers conducted the selection, evaluation, and data extraction from the literature, with any disagreements being resolved through consultation with a third researcher. The extracted information included essential details such as the first author, publication year, study location, study design type, patient numbers in each group, patient age, clinical classification, and follow-up duration. The evaluation of clinical efficacy indicators encompassed the number of recurrences and lymph node yield among postoperative patients, while safety indicators involved recording intraoperative complications, total complications, and abnormal ejaculation among patients. Perioperative indicators, on the other hand, comprised operative time, estimated blood loss, blood transfusion rate, and length of hospital stay. To assess randomized controlled trials (RCTs), both researchers independently evaluated the methodological quality using the Jadad scale. Likewise, non-randomized controlled studies (NRS) were appraised based on the Methodological Index of Non-Randomized Studies (MINORS) tool (17, 18). The MINORS tool contains a total of 12 items for the comparative studies, and each item is scored 0 to 2 points (0 = not reported; 1 =reported but insufficient information; 2 = reported and provided sufficient information), and based on the resulting methodological quality scores, the articles were categorized as having a low (>17), medium (≥10 and ≤17), or high risk of bias (<10) (18).

### Statistical analysis

Statistical analysis was conducted employing Review Manager 5.3 software to conduct the meta-analysis. Continuous variables were represented as mean ± standard deviation (SD) and assessed using mean difference (MD) with a 95% confidence interval (CI). Some studies reported clinical outcome measures as median or interquartile ranges. In order to facilitate analysis, means and SDs were derived using appropriate calculators based on sample size, median range, or interquartile range. For dichotomous outcomes, the odds ratio (OR) and its corresponding 95% CI were utilized as statistical indicators. Heterogeneity across studies was evaluated using the Chi-squared (χ2) and I-squared (I^2^) tests. When P > 0.1 and I^2^ < 50%, it was considered indicative of minor heterogeneity, thus employing a fixed effects model for data analysis. Conversely, when P ≤ 0.1 and I^2^ > 50%, significant heterogeneity was observed, necessitating the adoption of a random effects model. Subgroup and sensitivity analyses were carried out as needed to identify potential sources of heterogeneity. Statistical significance was set at P<0.05.

## Results

### Characteristics of the included studies

**Figure 1:**
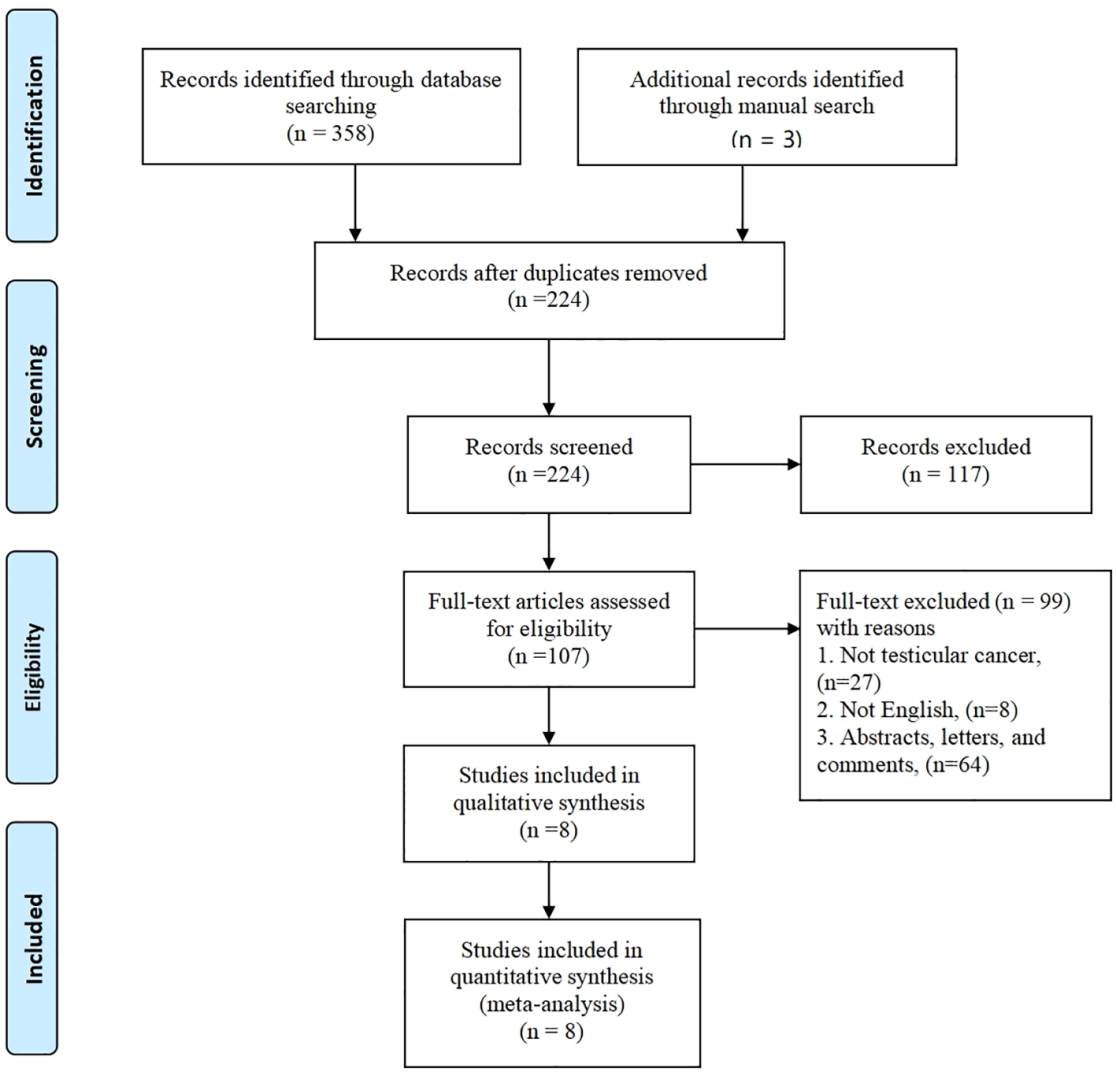
Flow of studies through the review.

The initial database search yielded a total of 358 articles, while an additional 3 articles were sourced through a manual search, bringing the overall count to 361. Out of these, 134 articles were found to be duplicates. Subsequently, 117 articles were excluded based on a review of their titles or abstracts, resulting in 107 articles that underwent further evaluation. After a comprehensive review of the complete texts, an additional 99 articles were excluded. Ultimately, a total of 8 articles (19–26) met the criteria for inclusion in the meta-analysis. These selected articles encompassed 453 patients in the R-RPLND group and 3422 patients in the NR-RPLND group. The screening process is presented in **Figure 1**, illustrating the flow of literature selection. Detailed information about the included studies is provided in **Table 1**. The quality assessment of the included studies is outlined in **Table 2**. The mean MINORS score was calculated to be 17.13 ± 1.81, indicating high evidence quality among the included studies. Notably, none of the included studies were randomized controlled trials (RCTs), so the Jadad scale was not applicable.

**Table 1:** Characteristics of studies included in the analysis.

Author	Region	Year	Study design	R-RPLND (N)	NR-RPLND (N)	Clinical stage	Primary/Post-chemotherapy	Matching/comparable variables	Conversion (N)	Follow-up (months)
Acar, O. et al. (19)	Turkey	2014	OCS(R)	8	10	IIA-III	Both	1,3,4,5,6,8,9,11	1	9.3 ± 4.9
Bhanvadia, R. et al. (20)	USA	2021	OCS(R)	44	319	NA	Primary	1,2,6,8	NA	NA
Brown, CT. et al. (21)	USA	2020	OCS(R)	269	2851	NA	Both	1,6	NA	NA
Grenabo Bergdahl, A. et al. (22)	Sweden	2022	OCS(P)	27	58	≥ IIA	Both	1,2,3,4,5,6,8,9,11	1	23
Harris, KT. et al. (23)	USA	2015	OCS(R)	16	21	I	Primary	1,2,4,5,8,9,10	2	2.8/13.5
Li, R. et al. (24)	USA	2021	OCS(R)	30	63	IIa- IIIc	Post-chemotherapy	1,2,3,4,5,7,8,9,11	NA	18.2
Lloyd, P. et al. (25)	UK	2022	OCS(R)	28	72	NA	Both	2,3,4,5,6,7,8,9,10,11	NA	36/60
Xu, Y. et al. (26)	China	2021	OCS(R)	31	28	I	Primary	1,2,4,5,6,7,8,9,10,11	NA	24/68.5

R-RPLND, Robotic retroperitoneal lymph node dissection; NR-RPLN, Non-Robotic retroperitoneal lymph node dissection; OCS, observational clinical study; P, prospectively collected data; R, retrospectively collected data; Matching/comparable variables: 1 = age, 2 = BMI, 3 = Size of mass, 4 = Operative time, 5 = Estimated blood loss, 6 = Length of stay, 7 = Transfusion, 8 = Major complications, 9 = Lymph node yield, 10 = Ejaculatory disorders, 11 = Recurrence; NA, data not available.

**Table 2:** Risk of bias for the involved studies.

Methodological Items for non-randomized studies	Acar, O. et al. (19)	Bhanvadia, R. et al. (20)	Brown, CT. et al. (21)	Grenabo Bergdahl, A. et al. (22)	Harris, KT. et al. (23)	Li, R. et al. (24)	Lloyd, P. et al. (25)	Xu, Y. et al. (26)
Clearly Stated Aim	2	2	2	2	2	2	2	2
Consecutive Patients	2	2	2	2	2	2	2	2
Prospective Data Collection	2	0	2	1	1	2	2	0
Appropriate Endpoint	2	2	2	2	2	2	2	2
Unbiased Endpoint Assessment	0	0	0	0	0	0	0	0
Appropriate Follow-Up	2	0	0	2	2	2	2	2
Loss to Follow-Up <5%	1	0	0	1	1	1	1	1
Prospective Study Size Calculation	0	0	0	2	0	0	0	0
An adequate control group	2	2	2	2	2	2	2	2
Contemporary groups	2	2	2	2	2	2	1	2
Baseline equivalence of groups	1	2	1	1	2	2	1	2
Adequate statistical analyses	2	2	2	2	2	2	2	2
Score	18	14	15	19	18	19	17	17

The quality of NRS was evaluated with the MINORS.

### Total operative time

**Figure 2:**
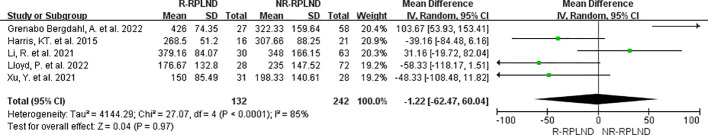
Forest plot of R-RPLND versus NR-RPLND on total operative time.

The total operative time was examined and compared in five studies (22–26). A random effects model was employed to combine the analyses (I^2 = ^85%,P<0.0001). The findings revealed no statistically significant disparity in operative time between the two groups (MD = -1.22; 95% CI -62.47 to 60.04; P = 0.97) (**Figure 2**).

### Amount of blood loss

**Figure 3:**
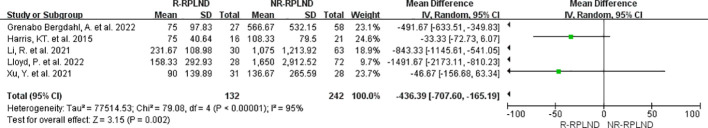
Forest plot of R-RPLND versus NR-RPLND on amount of blood loss.

The estimated blood loss reported in the five studies (22–26) was analyzed through meta-analysis. A random effects model to combine the data (I^2 = ^95%,P<0.00001). The findings indicated a statistically significant difference in estimated blood loss between the R-RPLND and NR-RPLND groups (MD = -436.39; 95% CI -707.60 to -165.19; P = 0.002) (**Figure 3**), with a reduction in blood loss in the R-RPLND group.

### Transfusion rate

**Figure 4:**
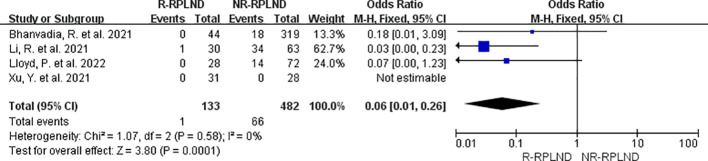
Forest plot of R-RPLND versus NR-RPLND on transfusion rate.

The transfusion rates reported in the four studies (20, 24–26) were subjected to thorough meta-analysis. A fixed effects model in the combined analysis (I^2 = ^0%,P = 0.58). The findings demonstrated a statistically significant difference in transfusion rates between the two groups (OR = 0.06; 95% CI 0.01 to 0.26; P = 0.0001) (**Figure 4**), indicating a decrease in the R-RPLND group compared to the NR-RPLND group.

### Hospital stay

**Figure 5:**
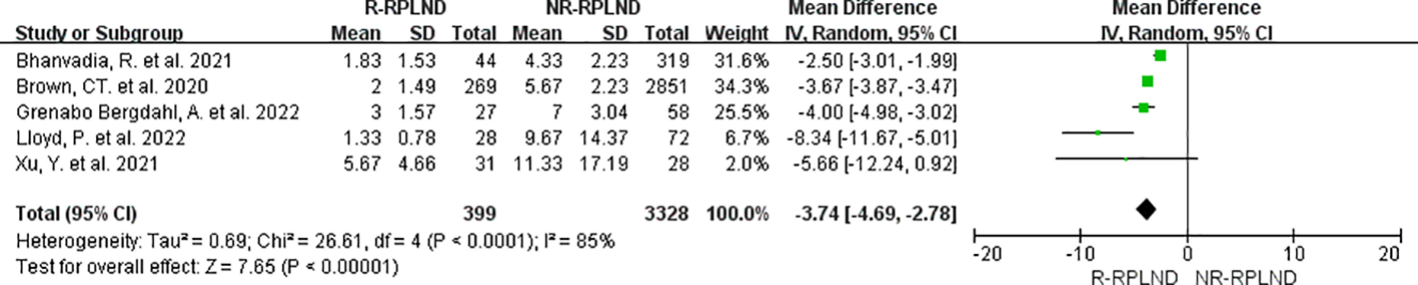
Forest plot of R-RPLND versus NR-RPLND on hospital stay.

The five studies (20–22, 25, 26), which reported and compared length of stay, were subjected to meta-analysis. A random effects model was employed to merge the findings (I^2 = ^85%,P<0.0001). Subsequent analysis divulged a statistically significant disparity in the length of stay between the two groups (MD=-3.74; 95% CI -4.69 to -2.78; P<0.00001) (**Figure 5**), illustrating a decrease in the duration of hospitalization among individuals in the R-RPLND group as opposed to those in the NR-RPLND group.

### Oncologic outcomes

**Figure 6:**
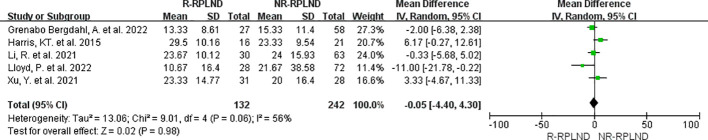
Forest plot of R-RPLND versus NR-RPLND on the mean lymph node yield.

The combined data from five studies (22–26) involving 374 patients (132 in the R-RPLND group and 242 in the NR-RPLND group) were analyzed in meta-analysis to determine the mean lymph node yield. A random effects model was employed to merge the results (I^2 = ^56%,P = 0.06). The findings of the meta-analysis indicated that there was no statistically significant difference in lymph node yield between the R-RPLND and NR-RPLND groups (MD = -0.05; 95% CI -4.40 to 4.30; P = 0.98) (**Figure 6**).

### Complications

The classification of surgery-related complications was carried out utilizing the widely accepted Clavien-Dindo system (27). We categorize complications into two groups based on their severity: those falling under grades II or lower and those of grade III or higher. In order to assess the overall incidence of complications in each cohort, the total complication rate was evaluated.

**Figure 7:**
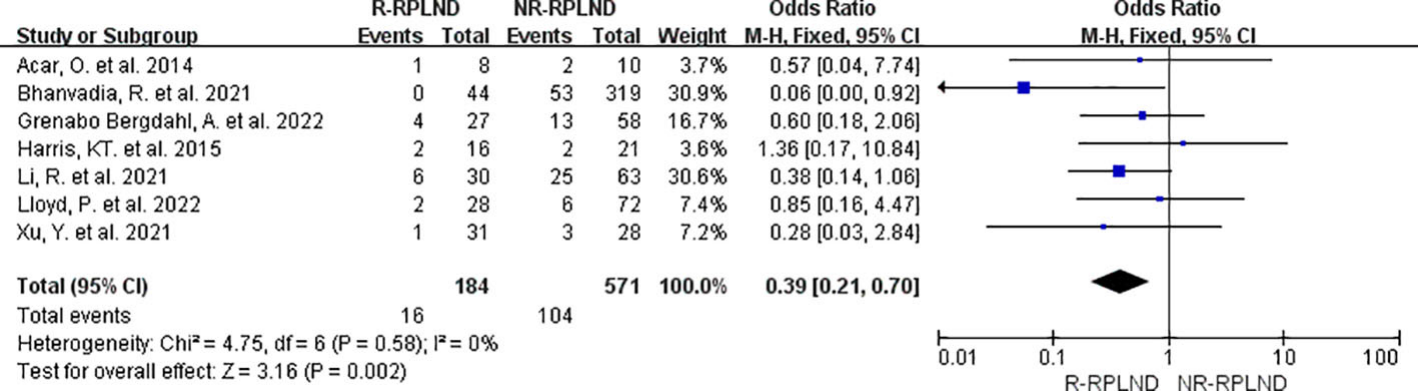
Forest plot of R-RPLND versus NR-RPLND on the overall postoperative complication rate.

A meta-analysis was conducted on the seven studies (19, 20, 22–26) that provided data on postoperative complication rates and made comparisons between groups. A fixed effects model for the combined analysis (I^2 = ^0%,P = 0.58). The meta-analysis findings revealed a statistically significant disparity in total complication rates between the two groups (OR = 0.39; 95% CI 0.21 to 0.70; P = 0.002) (**Figure 7**). As a result, it was demonstrated that the R-RPLND group exhibited a significantly lower incidence of total complications than the NR-RPLND group.

**Figure 8:**
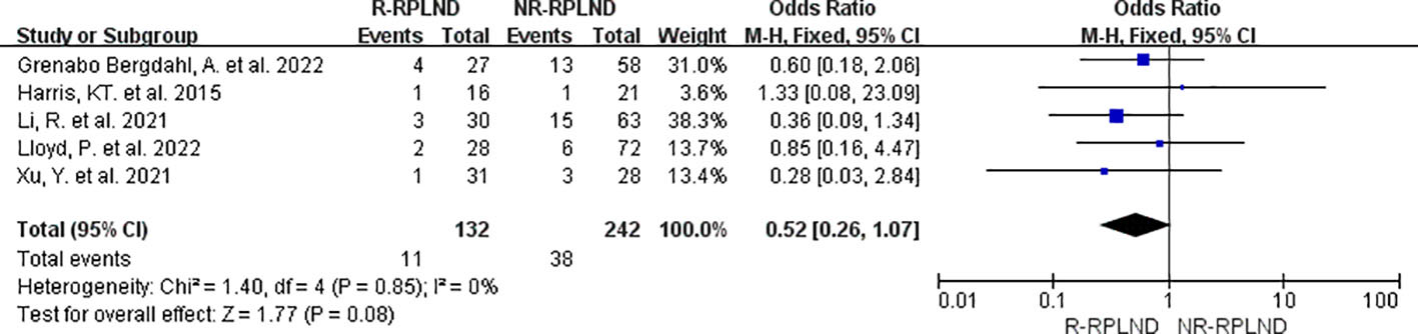
Forest plot of R-RPLND versus NR-RPLND on the surgical complication according to Clavien-Dindo grade ≥ III.

For the evaluation of surgical complications classified as Clavien-Dindo grade ≥ III, a meta-analysis was conducted using the data from five studies (22–26) that reported and compared the incidence of such complications. A fixed effects model for the combined analysis (I^2 = ^0%,P = 0.85). The results of the meta-analysis indicated no statistically significant distinction between the two groups in terms of the incidence of surgical complications for Clavien-Dindo grade ≥ III (MD = 0.52; 95% CI 0.26 to 1.07; P = 0.08) (**Figure 8**). These findings suggest no substantial variation in the occurrence of surgical complications of this severity between the R-RPLND and NR-RPLND groups.

**Figure 9:**
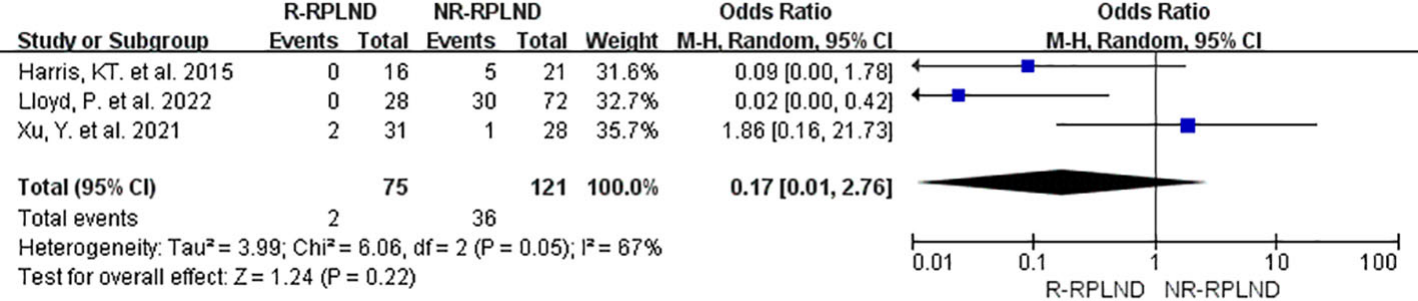
Forest plot of R-RPLND versus NR-RPLND on the postoperative ejaculation disorders.

In order to assess the incidence of postoperative abnormal ejaculation, a meta-analysis was conducted on the data from three studies (23, 25, 26) that reported and compared this outcome. A random effects model for the combined analyses (I^2 = ^67%,P = 0.05). The findings of the meta-analysis indicated no statistically significant distinction in the incidence of postoperative abnormal ejaculation between the R-RPLND and NR-RPLND groups (OR = 0.17; 95% CI 0.01 to 2.76; P = 0.22) (**Figure 9**). These results suggest no substantial variation in the occurrence of this specific complication between the two treatment groups.

### Recurrence rate

**Figure 10:**
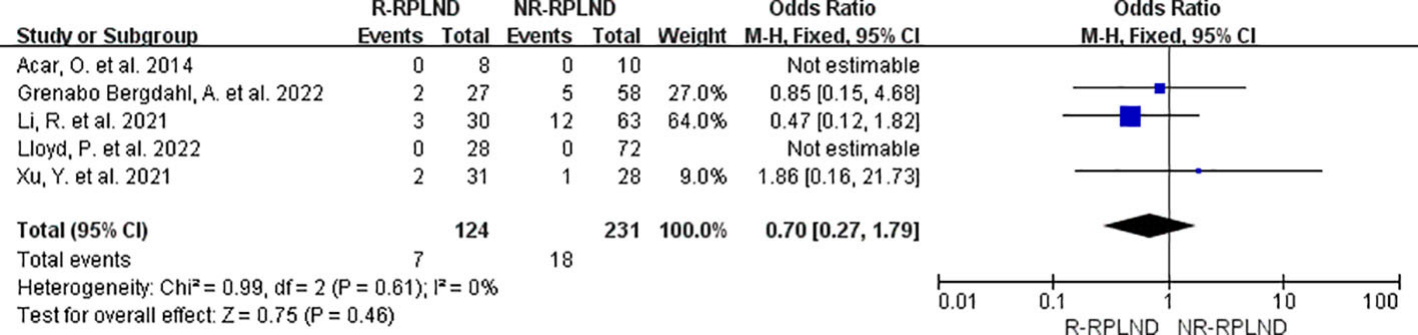
Forest plot of R-RPLND versus NR-RPLND on the postoperative recurrence rate.

Five studies (19, 22, 24–26) reporting and comparing postoperative recurrence rates were meta-analyzed. A fixed-effects model was applied to combine the analyses (I^2 = ^0%,P = 0.61). The results showed that the difference in postoperative recurrence rates between the two groups was not statistically significant (MD = 0.70; 95% confidence interval 0.27 to 1.79; P = 0.46) (**Figure 10**).

### Sensitivity analysis and subgroup analysis

Meta-analysis revealed high heterogeneity in the outcome indicators of operative time, estimated blood loss, length of hospital stay, mean lymph node yield, and abnormal postoperative ejaculation, and sensitivity analysis was performed by excluding individual studies one by one from the outcome analysis, with operative time (excluding the study by Grenabo Bergdahl, A (22), length of hospital stay (excluding the study by Bhanvadia, R (20), mean lymph node yield (excluding the study by Harris, KT (23) and abnormal postoperative ejaculation (excluding the study by Xu, Y (26) were significantly less heterogeneous (**Table 3**).

**Table 3:** The results of sensitivity analysis.

Indicators	MD/OR	P	I²
Sensitivity analysis			
Total operative time	-27.12[-67.53,13.28]	0.08	56%
Hospital stay	-4.27[-5.38,-3.16]	0.04	64%
Mean lymph node yield	-1.42[-4.42,1.57]	0.20	35%
Postoperative ejaculation disorders	0.05[0.01,0.36]	0.52	0

To indicate the total postoperative complication rate, we performed a subgroup analysis according to surgical modality (RA-RPLND versus O-RPLND/L-RPLND). The results showed that O-RPLND was consistent with previous results (OR = 0.36; 95% CI 0.19 to 0.68; P = 0.002). However, the results following the L-RPLND subgroup analysis contradicted the previous results, with no statistically significant difference in the total postoperative complication rate between the RA-RPLND and L-RPLND groups (OR = 0.64; 95% CI 0.15 to 2.78; P = 0.55).

## Discussion

In summary, this meta-analysis involving eight studies (19–26) aimed to compare the safety and efficacy of R-RPLND and NR-RPLND in managing early NSGCT. The results showed that R-RPLND and O-RPLND/L-RPLND were safe and effective for retroperitoneal lymph node dissection. Compared to NR-RPLND, R-RPLND significantly reduced surgical blood loss, decreased total postoperative complication rate, shorter hospital stay, and faster patient recovery, and should be considered a viable alternative to O-RPLND/L-RPLND. In addition, there were no statistical differences between the two groups regarding perioperative and oncological outcomes regarding total operative time, the incidence of postoperative complications grade≥III, abnormal ejaculation rate, lymph node yield, and postoperative recurrence rate.

For patients with early NSGCT, there are three treatment options following surgical orchiectomy and biopsy: active surveillance, chemotherapy, and RPLND. It is crucial that surveillance is appropriate for a subset of patients with stage I NSGCT who can be successfully treated by surgical resection (28). Chemotherapy is effective, but the side effects of chemotherapeutic agents and the risk of cardiovascular disease and secondary tumors are challenging to overcome (29). Currently, RPLND provides accurate staging, is the effective method for detecting these lesions, and provides a 95% good prognosis (30).Perioperative complications are the main drawbacks of the open RPLND surgical approach, such as long operative time, high intraoperative bleeding, high chance of postoperative complications, extended hospital stay, and ease of recurrence (31, 32). The robotic surgical system exhibits remarkable stability, effectively eliminating the minor hand tremors that can occur during manual procedures. Furthermore, the robotic endoscopic wrist possesses superior flexibility, enabling access to narrow and otherwise inaccessible spaces beyond the human hand’s reach. Consequently, applying this advanced technology in minimally invasive retroperitoneal lymph node dissection procedures results in reduced trauma minimized bleeding, and a decreased incidence of postoperative complications (33). A comprehensive analysis was conducted using data from the largest open inpatient care database in the United States, which compared the outcomes of 44 robotic-assisted Retroperitoneal Lymph Node Dissections (R-RPLNDs) with 319 open RPLNDs (O-RPLNDs) (20). The study revealed notable advantages in favor of R-RPLND, with significantly lower rates of bowel obstruction (0.0% vs. 8.4%), genitourinary complications (0% vs. 7.2%), and the need for blood transfusion (0% vs. 5.6%) compared to O-RPLNDs. Moreover, patients who underwent O-RPLND had a median length of stay more than twice as long as those who underwent R-RPLND, with a median of 1.5 days (IQR: 1-3) versus 4 days (IQR: 3-6) respectively. These findings underscore the favorable outcomes and reduced postoperative complications associated with R-RPLND compared to the conventional open approach. The examination conducted by Cheney et al. (34) encompassed a cohort of 18 patients, 8 of whom underwent Retroperitoneal Lymph Node Dissection (RA-RPLND) following chemotherapy. Impressively, none of these 8 patients experienced any major complications throughout a median follow-up duration of 22 months. Furthermore, no instances of recurrence were observed, and an overwhelming majority (91%) of patients maintained normal ejaculatory function. Multiple studies have consistently demonstrated that robotic RPLND outperforms traditional approaches by significantly reducing perioperative complications (0% vs. 16.6%, p<0.01) (20). These results underscore the remarkable advantages of robotic RPLND in minimizing risks associated with the procedure, thereby establishing it as an optimal and minimally invasive alternative. In our research, a comprehensive analysis was conducted to compare the outcomes of Retroperitoneal Lymph Node Dissection (RPLND) using different surgical modalities, including Open RPLND (O-RPLND), Laparoscopic RPLND (L-RPLND), and Robotic-Assisted RPLND (RA-RPLND). The results demonstrated several significant advantages associated with RA-RPLND when compared to O-RPLND/L-RPLND. Specifically, RA-RPLND exhibited reduced estimated blood loss (MD = -436.39; 95% CI -707.60 to -165.19; P = 0.002), a lower transfusion rate (OR = 0.06; 95% CI 0.01 to 0.26; P = 0.0001), lower total complications (OR = 0.39; 95% CI 0.21 to 0.70; P = 0.002), and a shorter length of hospital stay (MD = -3.74; 95% CI -4.69 to -2.78; P < 0.00001). These findings align with previous studies that have reported similar outcomes. However, when we conducted subgroup analyses based on surgical modality, the findings remained consistent with previous results regarding O-RPLND (OR = 0.36; 95% CI 0.19 to 0.68; P = 0.002). Interestingly, the results for L-RPLND contradicted previous findings, as there was no statistically significant difference in total postoperative complication rates compared to RA-RPLND (OR = 0.64; 95% CI 0.15 to 2.78; P = 0.55). It is important to note that this inconsistency may be attributed to the inclusion of fewer studies in the L-RPLND subgroup analysis, which could potentially introduce a higher likelihood of false-positive or false-negative results. This highlights the need for further research and larger sample size to obtain more conclusive results in this particular subgroup.

Several studies have highlighted the potential limitations of the robotic-assisted retroperitoneal lymph node dissection (R-RPLND) technique. One primary concern centers around the extended duration of the robotic approach compared to the conventional open RPLND procedure. Contemporary investigations utilizing robotic methods have identified a median operative time ranging from 235 to 330 minutes, whereas open RPLND typically encompasses a timeframe of 132 to 214 minutes (7, 15). Nevertheless, our research findings indicate that the operative time between robotic and open/laparoscopic retroperitoneal lymph node dissection is comparable (MD = -1.22; 95% CI -62.47 to 60.04; P = 0.97). Another concern concerns the escalated costs incurred due to the increased operative time associated with robotic RPLND. Prolonged surgical procedures result in heightened expenditure on anesthesia (35). Additionally, the utilization of robotic surgery necessitates investments in specialized instruments, further contributing to the higher cost of robotic RPLND compared to open surgery. The majority of studies conducted to date have had follow-up durations of less than 24 months, thus offering limited insights into the long-term oncological effectiveness of R-RPLND, particularly when juxtaposed against the robust evidence supporting O-RPLND. Furthermore, considerable heterogeneity is observed in the number of lymph nodes extracted during R-RPLND procedures, with some instances recording as few as seven lymph nodes (36).

The study still showed several limitations: (1) different inclusion and exclusion criteria in the included studies, with significant differences between patients enrolled; (2) the included studies were all non-randomized controlled studies, which may have led to a high degree of heterogeneity in the results; (3) no subgroup analysis was performed for studies of pre- versus post-chemotherapy retroperitoneal lymph node dissection; (4) fewer studies comparing robotic and laparoscopic were included fewer; (5) significant heterogeneity in the results of some of the analyses, and attempts to perform sensitivity analyses and subgroup analyses were not able to fully identify the sources of heterogeneity.

## Conclusions

In the realm of testicular cancer management, there is promising potential for the utilization of R-RPLND. R-RPLND demonstrates advantageous attributes such as reduced blood loss, decreased overall surgical complications, and shorter hospitalization periods, facilitating quicker patient recovery when compared to patient-specific O-RPLND or L-RPLND. These factors render R-RPLND a safe and efficacious treatment option for testicular cancer patients, comparable to NR-RPLND. However, it is essential to acknowledge certain limitations within the existing body of research. The substantiating evidence for R-RPLND primarily stems from small-scale, single-institution studies that need more adequate comparisons with L-RPLND or O-RPLND. This inherent limitation raises concern regarding potential publication bias and necessitates cautious interpretation of the findings. To validate the current conclusions and establish a comprehensive understanding of R-RPLND’s role in testicular cancer treatment, further investigations employing larger sample sizes, prospective design, multiple centers, extended follow-up durations, and randomized controlled trials are warranted. In summary, while R-RPLND holds promise in the management of testicular cancer, several considerations deserve attention. Future research endeavors should confirm the current findings and enhance our knowledge regarding the long-term oncological efficacy and overall utility of R-RPLND.

## Data availability statement

The original contributions presented in the study are included in the article/supplementary material. Further inquiries can be directed to the corresponding author.

## Author contributions

YY: Conceptualization, Data curation, Formal analysis, Investigation, Methodology, Project administration, Software, Supervision, Validation, Writing – original draft, Writing – review & editing. DZ: Conceptualization, Data curation, Investigation, Methodology, Supervision, Writing – review & editing. YN: Conceptualization, Data curation, Investigation, Methodology, Software, Writing – review & editing. HL: Conceptualization, Data curation, Investigation, Methodology, Writing – review & editing. XQ: Conceptualization, Data curation, Investigation, Methodology, Writing – review & editing. YT: Conceptualization, Data curation, Investigation, Methodology, Writing – review & editing. YL: Conceptualization, Data curation, Investigation, Methodology, Writing – review & editing. XY: Conceptualization, Data curation, Investigation, Methodology, Resources, Supervision, Validation, Writing – review & editing.
